# A Bayesian calibration model for combining different pre-processing methods in Affymetrix chips

**DOI:** 10.1186/1471-2105-9-512

**Published:** 2008-12-01

**Authors:** Marta Blangiardo, Sylvia Richardson

**Affiliations:** 1 Centre for Biostatistics, Imperial College, St. Mary's Campus, Norfolk Place, London, W2 1PG, UK

## Abstract

**Background:**

In gene expression studies a key role is played by the so called "pre-processing", a series of steps designed to extract the signal and account for the sources of variability due to the technology used rather than to biological differences between the RNA samples. At the moment there is no commonly agreed gold standard pre-processing method and each researcher has the responsibility to choose one method, incurring the risk of false positive and false negative features arising from the particular method chosen.

**Results:**

We propose a Bayesian calibration model that makes use of the information provided by several pre-processing methods and we show that this model gives a better assessment of the 'true' unknown differential expression between two conditions. We demonstrate how to estimate the posterior distribution of the differential expression values of interest from the combined information.

**Conclusion:**

On simulated data and on the spike-in Latin Square dataset from Affymetrix the Bayesian calibration model proves to have more power than each pre-processing method. Its biological interest is demonstrated through an experimental example on publicly available data.

## Introduction

In gene expression studies, one of the first steps of the statistical analysis is to quantify the signal and correct the systematic noise through pre-processing, a series of actions designed to extract the signal and the sources of variability due to the technology used rather than to biological differences between the RNA samples. Many studies in the literature present the importance of pre-processing and show how this can influence the results in terms of differential expression (see, for example, [1] and [2]).

However, an agreed gold standard method does not exist and as Allison and colleagues [3] discuss in a recent paper, researchers are torn between the different pre-processing methods and usually end up restricting their analysis to using only one (often the most commonly used or the most user-friendly). A simple alternative strategy is to perform the analysis using two different pre-processing methods and then compare the results in terms of differential expression, focussing attention on the genes in the intersection.

The former strategy is reductive while the latter relies on the arbitrary choice of two methods and on that of considering only their intersection. Neither of these approaches makes optimal use of all the information provided by the pre-processing methods. As an alternative Hein and colleagues [4] and Turro and colleagues [5] proposed a method that, starting at the probe level, integrates all the pre-processing steps and their associated uncertainty in one single framework, and they demonstrated an increase in power compared to the methods most used in the literature.

A different strategy is proposed in this paper, as we aim at providing a method of analysis of a gene-expression experiment that combines and synthesises the information from several pre-processing methods, to obtain a better calibrated estimate of differential expression. In the context of microarray, many researchers have proposed different strategies to pool together several independent studies. In particular, they have focused attention on the integration of each gene effect across studies [6] or on the evaluation of the consistency of differential expression across platforms [7]. Conlon *et al*. [8,9] have proposed a different approach: they do not estimate a combined gene effect across studies, but rather evaluate its differential expression through a pooled binary indicator for independent studies performed on the same platform. Their model is formulated in a Bayesian perspective and the posterior probability of differential expression is the quantity of interest. Later, Scharpf *et al*. [10] have extended Conlon's work to include the comparison of several platforms and to focus attention also on genes discordant across the experiments through the estimate of the sign of differential expression for each experiment. These papers share a *meta-analytical *framework, devoting their interest to synthesise several independent studies. In a different perspective Yang *et al*. [11] proposed a model for combining several measures of differential expression on the same experiment into an index; then they ranked the genes accordingly, using a permutation based test to select the differentially expressed genes. Similarly, we work on a single experiment, but concentrate on combining several pre-processing techniques; thus we adopt a *measurement error *perspective assuming for each gene a latent (unmeasured) 'true' differential expression and for each pre-processing method: (i) a measured value that departs from it (bias) and (ii) a variance component.

Modelling measurement error is common practice in epidemiology, where errors in the recording of explanatory variables are a frequent problem that has to be taken into account during the statistical analysis (see for example [12-15]); the formulation in a Bayesian framework has been discussed in the early 1990s by Thomas *et al*. [16], Richardson and Gilks [17,18] and Richardson [19] among others, placing particular emphasis on the way their approach propagates coherently all sources of uncertainty in the data onto the estimation of the parameters of interest.

We follow Richardson and Gilks and specify a Bayesian calibration model for assessing differential expression in Affymetrix microarray, which combines the information from several pre-processing techniques and it is implemented using the freely available software WinBUGS [20]. The performance of the combined model for assessing differential expression is compared with the performance of the equivalent Bayesian model on each single pre-processing method using simulated data and the Latin square data set provided by Affymetrix [21]. The combined method is shown to have better operating characteristics; its biological interest is discussed on an experiment publicly available to evaluate the effect of High Fat Diet versus Normal Fat Diet in mice adipose tissue.

## Results

### Bayesian calibration model

A combined model for different pre-processing methods is characterised by two measurement error components: (i) a measure of the bias from the 'true' differential expression, that can assume additive or multiplicative form and (ii) a measure of variability around the mean gene expression.

When the bias is multiplicative, following the general formulation given in [22], we model the observed log expression value for gene *g *= 1, ... *G*, pre-processing method *j *= 1, ..., *J*, condition *k *= 1, 2 and replicate *r *= 1, ..., *R *as a Normal distribution:

(1) $$\begin{array}{cc} y_{gj1r}\sim N(\alpha_{gj}-\frac{1}{2}\times \delta_{g}\times \phi_{j},\sigma_{gj1}^{2}) & y_{gj2r}\sim N(\alpha_{gj}+\frac{1}{2}\times \delta_{g}\times \phi_{j},\sigma_{gj2}^{2}) \end{array}$$

The parameter *α*_*gj *_represents the global gene expression that is specific to the gene *g *and the pre-processing method *j*, whereas *δ*_*g *_is the 'true' (unknown) differential expression that we would like to capture for gene *g*. The method specific coefficient *φ*_*j *_quantifies the multiplicative bias of the method with respect to the latent quantity *δ*_*g*_.

When the bias is additive (1) becomes:

(2) $$\begin{array}{cc} y_{gj1r}\sim N(\alpha_{gj}-\frac{1}{2}\times \delta_{g}+\xi_{j},\sigma_{gj1}^{2}) & y_{gj2r}\sim N(\alpha_{gj}+\frac{1}{2}\times \delta_{g}+\xi_{j},\sigma_{gj2}^{2}) \end{array}$$

where *ϕ*_*j *_is replaced by *ξ*_*j*_.

**Figure 1:**
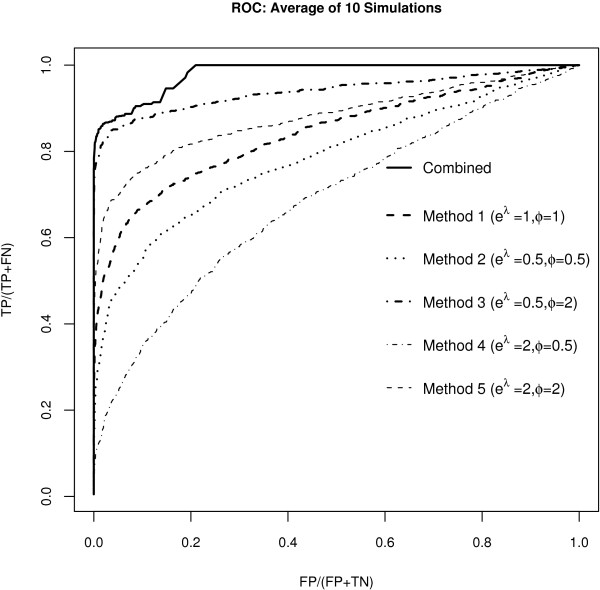
**ROC curve for the simulation study**. The plot shows the ROC curve for the Bayesian models averaged over the 10 simulated dataset: in each case we simulated 200 differentially expressed and 800 not differentially expressed genes. We have implemented the models either combining the five pre-processing methods together (solid line) or analysing each one separately and ranked the tail posterior probability of differential expression. The ROC curve for the combined model is above that of each pre-processing method, highlighting the benefit of using a combined model in terms of specificity and sensitivity.

**Figure 2:**
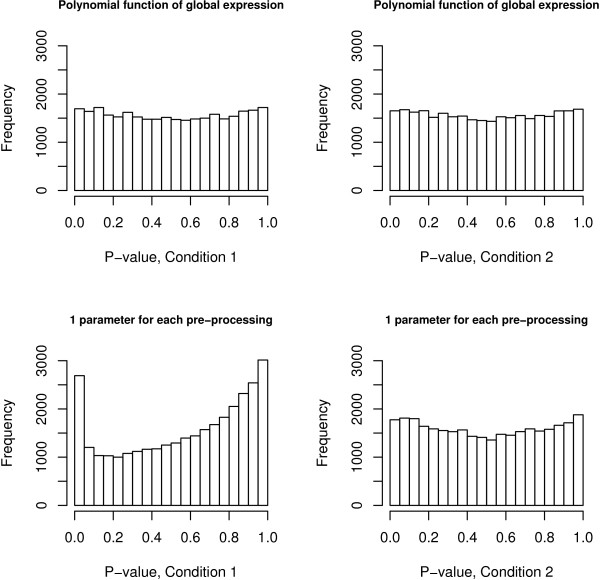
**Mixed Posterior Predictive checks for the Latin square data set**. The upper plots present the distribution of the mixed predictive p-values for the two conditions fitting the calibration model presented in (3) which assumes that the variability is specified by a polynomial function of the level of expression. They show a uniform behavior for both conditions, indicating an excellent model fit. The values for *λ*_1*jk*_, *λ*_2*jk *_and *λ*_3*jk *_are presented in Table 6 in Additional file 1. The bottom plots present the distribution of the p-values for the two conditions after fitting the simpler model assuming *λ*_2*jk *_= *λ*_3*jk *_= 0. They show a lack of fit, in particular for condition 1, indicating that the simple variance model is not appropriate for this dataset.

We empirically investigated the performance of additive and multiplicative bias on two datasets we use in the rest of the paper (see Materials and Methods for the description of the datasets). A pairwise comparison of the differential expression for the *J *methods is presented in Figure 1 and 2 of Additional file 1 and shows that the data are not lying on the diagonal, but are characterised by a slope different from 1. This suggests the presence of a multiplicative bias, while in general there is no evidence of a shift in the differential expression that would suggest the presence of an additive bias. For this reason, in the rest of the paper we consider the multiplicative bias model (1) and call *ϕ*_*j *_the relative bias as it indicates the inflation or deflation factor of the 'true' differential expression characteristic of a particular pre-processing method. Other details about the investigation we carried out on the two datasets are presented in the Discussion. In (1), for each gene, pre-processing and condition, the variance $\sigma_{gjk}^{2}$ is the result of a gene and condition specific component $\sigma_{gk}^{2}$ and an exponential error term specific to the pre-processing method and to the condition:

(3) $$\sigma_{gjk}^{2}=\text{exp}(\lambda_{1jk}+\lambda_{2jk}\times {\bar{y}}_{g}+\lambda_{3jk}\times {\bar{y}}_{g}^{2})\times \sigma_{gk}^{2}.$$

The exponential component is allowed to depend on the global expression of the gene $\left({\bar{y}}_{g}=\frac{1}{2JR}\sum_{j,k,r}y_{gjkr}\right)$ as it has often been noted that even after log transformation, the variability of the expression of a gene can be affected by its level of expression (see for example [23]). The use of a second order polynomial offers considerable flexibility yet involving a limited number of parameters; a simplified version of equation (3) can be formulated where the coefficients *λ*_2*jk *_and *λ*_3*jk *_are equal to 0, so that the exponential component becomes independent from the expression of the gene. Following [22] we assign a hierarchical structure on the probeset and condition specific component, to borrow strength from the entire set of genes:

(4) $$\sigma_{gk}^{2}\sim Ga^{-1}(a_{k},b_{k})$$

To complete the model we specify weakly informative prior distributions for all the remaining parameters as we do not have specific prior information available on these, so they will be informed by the data. To be precise, we specify a centred Normal distribution with large variance (10^5^) on *α*_*gj *_and *δ*_*g*_. The relative bias coefficients are modelled as *ϕ*_*j *_~*logN *(0, 10^5^) independently for *j *= 1, ..., *J*, imposing the identifiability constraint that ∏ *ϕ*_*j *_= 1.

The coefficients for the exponential component in (3) are assumed independent and to follow Normal distributions *λ*_1*jk *_~*N *(0, 10^5^), *λ*_2*jk *_~*N *(0, 10^5^), *λ*_3*jk *_~*N *(0, 10^5^), so that the function can model a wide variety of trends. As identifiability constraint, we impose that the sum of the experimental parameters in (3) over the three pre-processing methods is equal to 0 (Σ_*j *_*λ*_1*jk *_= 0, Σ_*j *_*λ*_2*jk *_= 0, Σ_*j *_*λ*_3*jk *_= 0). We use the exponential parametrization to ensure positivity of (3). Finally, *a*_*k *_~*Ga*(0.01, 0.01) and we model $1/\sqrt{b_{k}}\sim U[0,min_{g}{\left(s_{gk}^{2}\right)}^{-0.5}]$, where $s_{gk}^{2}$ is the sample variance for gene *g *and condition *k*; this choice ensures that the posterior distributions of *a*_*k *_and *b*_*k *_are proper and well adapted to the scale of the data, as justified in [24].

Models (1) and (3) allow the borrowing of information across genes to estimate *ϕ*_*j *_and *λ*_*jk*_, and across methods to estimate *δ*_*g *_and $\sigma_{gk}^{2}$. The hierarchical model specified by (1), (3), (4) and the prior distributions are estimated using an MCMC algorithm coded in WinBUGS [20] to simulate the prior/posterior distribution of all unknown parameters. More details can be found in the Materials and Methods section and in Additional file 1.

#### Tail posterior probability

For each gene we are interested in testing the hypothesis that the differential expression effect *δ*_*g *_is different from 0:

$$\begin{array}{ccc} H_{0}^{g}:\delta_{g}=0 & \text{vs} & H_{1}^{g}:\delta_{g}\neq 0 \end{array}$$

and a variety of decision rules based on the output of the hierarchical model can be constructed. We chose to use the tail posterior probability statistic introduced in [24] to measure the strength of the evidence against *H*_0_. This method considers a standardisation of the differential expression measure $z_{g}=\frac{\delta_{g}}{\sqrt{w_{g}}}$ where *w*_*g *_is a pooled measure of variability of *δ*_*g*_:

$$w_{g}=\frac{2}{RJ^{2}}\sum_{j=1}^{J}\left(\sigma_{gj1}^{2}+\sigma_{gj2}^{2}\right).$$

Here *R *is the number of replicates for each condition and *J *is the number of pre-processing methods considered. A value of *z*_*g *_is obtained at each iteration of the MCMC simulation after convergence is reached and the tail posterior probability statistic is then defined as follows:

(5) *p*(*z*_*g*_; *z*_*α*_) = *P*(|*z*_*g*_| > *z*_*α *_| **y**_g_)

where **y**_g _denotes all the data available for gene *g *and *z*_*α *_is the *α *quantile of the standard normal distribution (usually *α *= 0.05 and consequently *z*_*α *_= 1.96). As discussed in [24] the histogram of *p*(*z*_*g*_; 1.96) is characterised by a local peak on the right tail in the presence of differentially expressed genes; this peak can be used to define a reasonable cut off for the differential expression (see the section that describes the results on the experimental data for an illustrative example).

The tail posterior probability statistic can be loosely interpreted as a Bayesian analogy of the t-test. It makes full use of the Bayesian output being a function of the differential expression (*δ*_*g*_) and of the variability $\left(\sigma_{gj1}^{2}\text{ and }\sigma_{gj2}^{2}\right)$, is easy to use and was shown to have good statistical properties (see [24] for more details).

#### Assessing the fit of the model

Before using the calibration model as specified in (1)–(4) to infer differentially expressed genes, it is important to assess its ability to capture the sources of variability of the pre-processing methods, and in particular whether the parametrisation used in (3) is appropriate. One of the added benefits of working in a Bayesian framework is the ability to perform model checks by means of the predictive distribution of the parameters of interest. We use Mixed Posterior Predictive checks [25,26], applied on gene expression data by Lewin *et al*. [22,27] and focus attention on checking the gene specific variance, characterised by a hierarchical structure as described in equations (3) and (4). For each method, we compare the observed sample variance, calculated for the expression values, and the variance of the predicted expression values of each gene under the model using an empirical p-value.

Under the null hypothesis of the model being true, the distribution of the p-values should be approximatively uniform, while a poor model fit is indicated by departure from uniformity in the plot, suggesting a systematic difference between the observed values and the predicted ones (see Additional file 1 and [22,27] for more details).

The Mixed Posterior Predictive check is characterised by a certain degree of subjectivity as the researcher has to visually assess the fit of the model through a plot. Complementary to this, we also provide a quantitative measure of model fit, enabling a more direct comparison between different models by means of the Deviance Information Criterion (DIC) [28]. This index has been proposed as an extension of the Akaike Information Criterion when dealing with Bayesian hierarchical models. It is defined as a function of the deviance of the model and of the effective number of parameters included:

*DIC *= *E*_*θ *_[*D*(*θ*)] + *p*_*D*_

where *E*_*θ *_[*D*(*θ*)] is the posterior mean of the deviance of the model and *p*_*D *_is the effective number of parameters. When comparing two or more models, the one characterised by the smallest DIC shows the best fit to the data in hand.

### Performance on simulated data

To first evaluate the benefits of using a model that combines several pre-processing methods we simulated log expression values for 1000 genes, 5 pre-processing and 2 conditions, following the approach described in the section Materials and Methods. We set 200 genes as differentially expressed and the remaining 800 genes as not differentially expressed. We considered a simplification of equation (3) assuming (i) the same variance for the two conditions $\left(\sigma_{gj1}^{2}\equiv \sigma_{gj2}^{2}\right)$ and (ii) the exponential component as independent from the global gene expression (*λ*_2*jk *_= 0, *λ*_3*jk *_= 0). This does not detract from evaluating the comparative performance of the calibration model versus each method. To evaluate the consistency of our results we repeated the simulation 10 times and averaged the results.

The typical behavior of the combined method compared to each single pre-processing is presented in Figure 1: the ROC curve averaged over the 10 runs shows a greater sensitivity and specificity for the model that combines the five pre-processing approaches.

Ranking the pre-processing methods based on the ROC curve, Method 3, characterised by large relative bias and small variability (in the simulation set up *ϕ*_3 _= 2 and exp(*λ*_3_) = 0.5), shows the best performance, while on the other end, Method 4, characterised by small relative bias and high variability (in the simulation set up *ϕ*_4 _= 0.5 and exp(*λ*_4_) = 2), shows the worst ROC curve. Note that the 'reference' method, characterised by exp(*λ*_1_) = 1 and *ϕ*_1 _= 1 shows an average specificity and sensitivity.

The influence of the relative bias coefficient *ϕ*_*j *_can be evaluated when doing pairwise comparisons of methods with the same variability (Method 2 vs Method 3 and Method 4 vs Method 5). If it is greater than 1 (Method 3 and Method 5), the corresponding pre-processing methods are assumed to magnify the 'true' differential expression. This results in a stronger signal and consequently in a greater ability to discern true positives and true negatives.

On the other hand, comparing methods characterised by the same value for the relative bias coefficient (Method 2 vs Method 4 and Method 3 vs Method 5) the difference in their performance is explained by the exponential component of variability exp(*λ*_*j*_): a low value (Method 2 and Method 3) results in a higher precision of the estimates, enhancing the performance of the method in terms of specificity and sensitivity. Table 1 presents the operating characteristics for all the pre-processing methods and for the combined strategy. As we set 200 genes as differentially expressed in the simulation scenario, we consider the first 200 genes ranked according to the tail posterior probability (5) and evaluate the number of False Positives (FP), False Negatives (FN), True Positives (TP) and True Negatives (TN). The results are averaged over 10 repeats. The combined method is able to detect the maximum number of truly differentially expressed genes (179) and is characterised by only the 2.6% of False Positives. As already pointed out, the methods with a *ϕ*_*j *_> 1 (Method 3 and Method 5) have a higher signal, leading to a better performance, characterised by a small percentage of False Positives and False Negatives.

**Table 1:** Operating characteristics for simulated data

	DE	Non DE	FP (%)	TN (%)	TP (%)	FN (%)	sd
Combined	200	800	21 (2.6)	779 (97.4)	179 (89.5)	21 (10.5)	5.0
Method 1 (*exp*(*λ*) = 1, *ϕ *= 1)	200	800	61 (7.6)	739 (92.4)	139 (69.5)	61 (30.5)	5.5
Method 2 (*exp*(*λ*) = 0.5, *ϕ *= 0.5)	200	800	80 (10.0)	720 (90.0)	120 (60.0)	80 (40.0)	4.8
Method 3 (*exp*(*λ*) = 0.5, *ϕ *= 2)	200	800	26 (3.2)	774 (96.8)	174 (87.0)	26 (13.0)	4.1
Method 4 (*exp*(*λ*) = 2, *ϕ *= 0.5)	200	800	122 (15.2)	678 (84.8)	78 (49.0)	122 (61.0)	3.3
Method 5 (*exp*(*λ*) = 2, *ϕ *= 2)	200	800	45 (5.6)	755 (94.4)	155 (77.5)	45 (22.5)	5.8

The table presents the number of False Positives (*FP*), True Negatives (*TN*), True Positives (*TP*) and False Negatives (*FN*) in the first 200 genes ranked accordingly to their tail posterior probability. Note that *FP *= *FN *since the size of the list of differentially expressed genes is equal to the number of *true positives*. The combined method shows the smallest number of *FP *and *FN*. Out of the 5 pre-processing methods, the one characterised by a relative bias parameter *ϕ*_*j *_> 1 and a variability parameter *exp*(*λ*_*j*_) < 1 (Method 3) shows the best performance, due to the combination of high signal and low variability around the mean gene expression. The standard deviation calculated on the 10 runs is also reported in the table. Note that as FP, FN, TP and TN are a linear function of each other given a fixed sum, the standard deviation is the same for the 4 variables.

### Latin square data set

We applied the model presented in (1), (3) and (4) to the 10621 probesets present in the Latin square data set (experimental condition 2 versus 1), where only 64 genes are *truly differentially expressed*. The p-values histograms for the two conditions obtained from the Posterior Predictive checks are presented in the upper plots of Figure 2 and are characterised by a uniform behavior, suggesting a good model fit to the data. As a point of comparison we ran the simplified model characterised by *λ*_2*jk *_= *λ*_3*jk *_= 0 and present the corresponding Posterior Predictive checks in the lower plots of Figure 2. These plots show a clear deviation from uniformity with an over representation of small and large p-values, in particular for condition 1, suggesting a lack of flexibility of the simpler model to account for all the variability present in the data. Hence we retain the variance model in (3) for our analysis.

Table 2 presents a synthesis of the results for the combined model and for each single pre-processing method. We evaluated the operating characteristics of each method based on the first 64 probesets ranked accordingly to their tail posterior probability. The combined method shows an improvement in sensitivity and specificity, even if modest: out of the first 64 ranked probesets only 12 are false positives, while the number increases to 14 for RMA and then jumps to 23 and 31 for MAS5 and dChip respectively. When the gene list size increases, the performance for the different methods tends to converge.

**Table 2:** Operating characteristics for Latin Square dataset

	First 64 ranked probesets			
	FP (%)	TN (%)	TP (%)	FN (%)

Combined	12 (0.1)	10545 (99.9)	52 (81.3)	12 (18.7)
MAS5	23 (0.2)	10534 (99.8)	41 (64.1)	23 (35.9)
RMA	14 (0.1)	10543 (99.9)	50 (78.1)	14 (21.9)
dChip	31 (0.4)	10526 (99.6)	33 (51.6)	31 (48.4)

The table presents the operating characteristics of the combined method and of each pre-processing method on the first 64 probesets ranked accordingly to their tail posterior probability. The combined strategy is more able to recognize true positives and true negatives than each single method. Note that *FP *= *FN *since the size of the list of differentially expressed probesets is equal to the number of *true positives*.

Table 3 reports the posterior mean of the relative bias effect *ϕ*_*j *_together with the 95% credibility interval. We see that MAS5 and RMA are characterised by a mean value greater than 1, meaning that they inflate the estimate *δ*_*g *_relative to dChip.

**Table 3:** Posterior mean and credibility interval for *ϕ*_*j*_

	Latin Square data set		Real Experiment: HFD vs NFD	
	*E*(*ϕ*_*j *_| **y**)	*CI*95%	*E*(*ϕ*_*j *_| **y**)	*CI*95%

MAS5	1.382	[1.345–1.417]	1.449	[1.438–1.462]
RMA	1.144	[1.124–1.165]	1.064	[1.056–1.074]
dChip	0.632	[0.623–0.644]	0.648	[0.643–0.653]

Posterior mean and 95% credibility intervals for the relative bias effect *ϕ*_*j *_in the Latin Square data set (left) and in the experimental data analysis that compares the effect of high fat diet (HFD) and normal fat diet (NFD) on adipose tissue of mice (right).

Similar results in terms of parameter estimates and performance are obtained for additional pairwise comparisons on this dataset, as presented in Tables 1, 2, 3 of Additional file 1. These comparisons have been selected to represent a wide spectrum of differential expression and to show that the results are consistent across different experiments.

### Biological example: High Fat Diet versus Normal Fat Diet in mice adipose tissue

**Figure 3:**
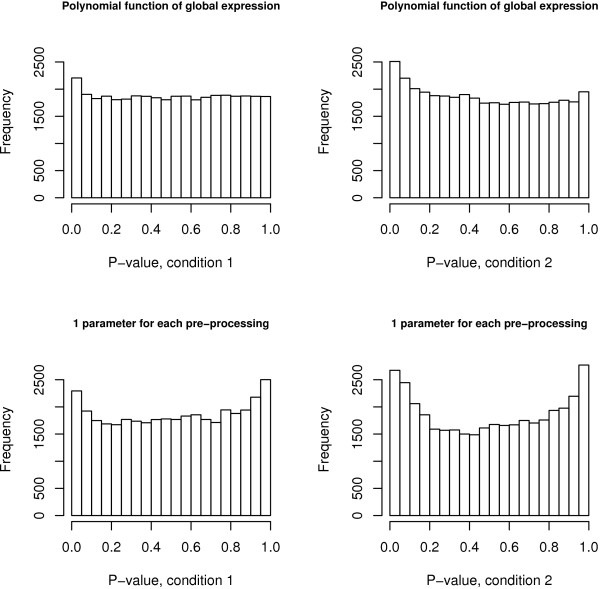
**Mixed Posterior Predictive checks for the HFD example**. The upper plots present the distribution of the p-values for the two conditions (HFD and NFD) after fitting the calibration model presented in (3) which assumes a polynomial function of the level of expression. They show a uniform behavior for both conditions, indicating good model fit. The values for *λ*_1*jk*_, *λ*_2*jk *_and *λ*_3*jk *_are presented in Table 7 of Additional file 1. The bottom plots present the distribution of the p-values for the two conditions (HFD and NFD) fitting the simpler calibration model assuming *λ*_2*jk *_= 0 and *λ*_3*jk *_= 0. The histograms indicate the presence of two peaks corresponding to very small and very large p-values, suggesting a poor fit.

We applied the model presented in (1), (3) and (4) to the 12488 probesets in the experiment to study the effect of high fat diet (HFD) versus normal fat diet (NFD) on mice adipose tissue, as part of the DGAP project [29]). As for the Latin Square data set the Mixed Posterior Predictive checks show a good fit, while the simpler model with *λ*_2*jk *_= *λ*_3*jk *_= 0 is not adequate (see Figure 3).

We use the histogram of the tail posterior probability to identify a reasonable cut off for calling a gene differentially expressed. Contrary to what happens for each single pre-processing method, the histogram of the tail posterior probability for the combined model shows a local peak on the right tail of the distribution (see Figure 3 of Additional file 1), indicating more evidence of differential expression. As suggested in [24], we select a high cut off value, in our case equal to 0.98, corresponding to the local peak on the combined distribution, and obtained a list of 292 'top' probesets classified as differentially expressed by the combined method. If we fixed the same cut off on the tail posterior probability for each pre-processing method, we would obtain substantially smaller lists with only 20 probesets classified as differentially expressed by MAS5, 32 by RMA and 41 by dChip. Selecting a standard threshold such as 0.95 gives similar results in term of the size of the lists: the combined one is the largest, while MAS5 is characterised by the smallest. This highlights the typical gain of confidence provided by the combined analysis: many more probesets have high posterior probability of being differentially expressed in the combined model than if we proceeded for each method separately. In order to perform a further comparison between the methods we also consider the first 292 probesets ranked according to the tail posterior probability for each single method. Note that in doing so we lower the cut off on the tail posterior probability scale for each method (0.78 for MAS5, 0.79 for RMA and 0.83 for dChip), introducing more noise in the list.

**Figure 4:**
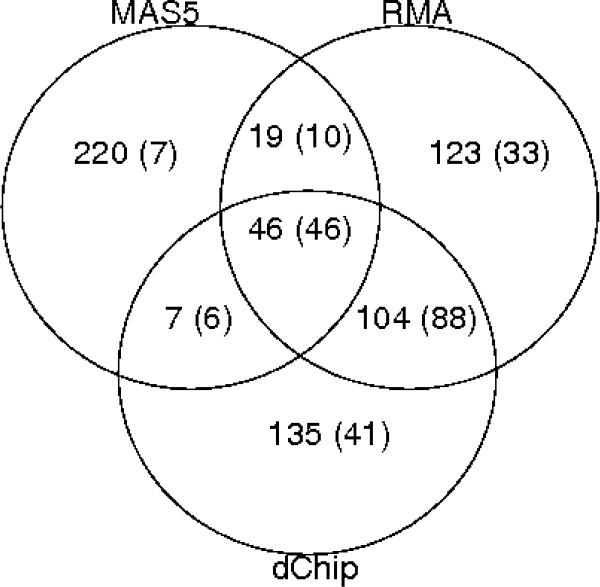
**Venn diagram**. The figure shows the number of probesets in the top list of 292 features for each method according to their relation with all the other methods. The number of probesets in common between 1,2 or all the methods and the combined one are shown in parenthesis. For instance, in the intersection of MAS5 and dChip there are 7 probesets; out of them 6 (shown in parenthesis) are in common also with the combined method.

Figure 4 shows the Venn diagram for the three single methods and the combined model. All the 46 probesets in the intersection of the three methods are included in the list of 'top' probesets by the combined model. Additionally, there are 61 probesets that are only found in the combined list and are of particular interest. These are characterised: (1) by a probability greater than 0.5 for at least two methods (usually RMA and dChip, or sometimes, MAS5 and one of the other two), (2) by a substantial different variability between the two conditions and a relatively small fold change (see Figure 4 in Additional file 1 for a boxplot of some of the 61 probesets). Considering each pre-processing method separately, these fold changes do not reach the top of the list, but the combined strategy increases their significance, by synthesising the evidence from the three pre-processing methods. This suggests that combining several pre-processing methods leads to larger lists of genes than considering the intersection of lists obtained for each method, as more information coming from different sources is taken into account.

**Figure 5:**
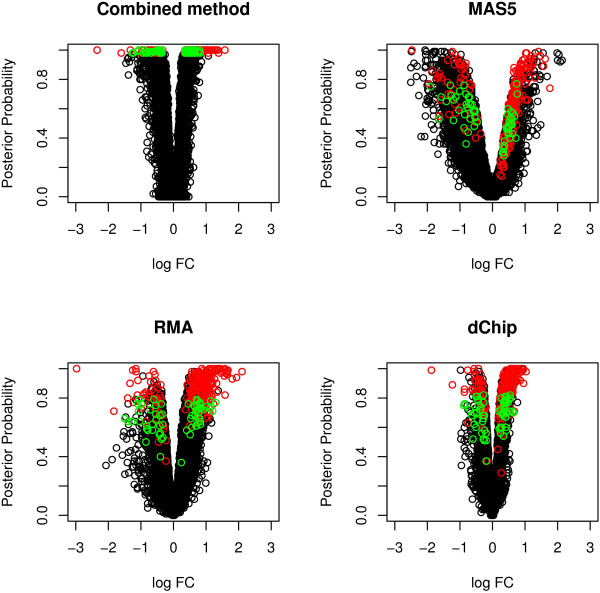
**Volcano plot for the HFD experiment**. The plot shows the different behavior of the pre-processing methods: dChip has a small variability resulting in a compact volcano plot, while MAS5 is characterised by a large variability that causes some probesets with very different values of the log fold change to have very similar posterior probability. The combined method shows a distribution close to dChip. The 292 probesets called differentially expressed by the combined model using a cut off of 0.98 on the tail posterior probability scale are highlighted in red in all the plots; the 61 probesets selected only by the combined method are highlighted in green in all the plots.

Figure 5 presents the plot of the log fold change versus the posterior probability (volcano plot) for the combined model and for each pre-processing method considered separately. The 292 probesets called by the combined method are highlighted in red and the 61 selected only by the combined approach are highlighted in green. The 292 probesets are placed in the upper half of the plot for RMA and dChip, being characterised by values of the posterior probability far from 0. This indicates that in general when a gene shows some evidence of differential expression for the two methods with smaller variability, the combined approach strengthens this evidence and places these probesets at the top of the list of differential expression. MAS5 is the method that contributes the least to the combined output, being associated with the largest variability. For this reason, some probesets with a tail posterior probability smaller than 0.5 for MAS5 can still be found at the top of the list for the combined method, if their posterior probability values for RMA and dChip are large enough. On the other hand, MAS5 shows the largest *ϕ*_*j *_effect (presented on the right hand side of Table 3); thus, as already observed in the previous sections, the method is characterised by a larger signal that may provide additional information on the differential expression. This results in 7 probesets called as differentially expressed only by MAS5 that are placed in the top list for the combined method. These probesets would not be selected as differentially expressed when considering only RMA and dChip.

The contribution of MAS5 to the combined output has been further investigated by implementing a version of our model that combines only RMA and dChip and comparing the results. In general, combining only these two methods results in 205 probesets classified as differentially expressed for a cut off of 0.98, again chosen on the basis of the local peaks on the tail posterior probability histogram (see Figure 5 of Additional file 1). Out of these, 163 are in common with the model combining the 3 methods and 42 are specific to the two-methods calibration model. In the three-methods combined model, these 42 probesets are either (i) borderline or (ii) far from the top of the list, characterised by high tail posterior probability for only one method. This shows that for some probesets there remains a non negligible source of uncertainty when only two methods are considered. When a third method is included, evidence in favor of the null hypothesis of no differential expression is added and these probesets are not classified as significant anymore. Moreover, including an additional method in the synthesis results in 129 new probesets called differentially expressed: these probesets are borderline in the model with only two methods, characterised by a posterior probability ranging between 0.81 and 0.96; for them the evidence against *H*_0 _is thus strengthened by the introduction of MAS5 in the model.

Table 4 presents the number of annotated genes in the list of the first 292 ranked probesets for the model combining the three pre-processing methods and for each single one. For the combined method the most represented biological processes are the metabolic ones, functions associated with the response of the body to a change in the diet. The number of genes involved in *cellular metabolism*, is 105 for the combined method, 70 for dChip, 87 for MAS5 and 88 for RMA. Similarly the number of genes involved in primary metabolism or macromolecular metabolism is larger for the combined method than for each pre-processing one.

**Table 4:** Annotation for differentially expressed genes

First 292 genes with the largest posterior probability				
	Combined Model	MAS5	RMA	dChip

Biological Processes	181	146	171	168
Molecular Functions	193	154	180	173
Cellular Components	179	145	173	169
KEGG pathways	223	182	215	205

The table presents the number of differentially expressed genes annotated by GO or KEGG considering the first 292 probesets with the largest tail posterior probability for each method and for the combined one. The most represented KEGG pathways are mainly related to immune response and oxidation (*Antigen processing and presentation*, *MAPK signalling pathway*, *PPAR signaling pathway*). For these pathways the number of genes found by the combined method is always larger than the one found by each pre-processing method. Moreover the combined model also calls 6 genes that are involved in the *Insulin signalling pathway*, specifically related to diabetes, one of the diseases recently highlighted to be associated with high fat diet in mice (see for example [45] and [46]). The same pathway is present with only 3 genes in MAS5 and with 2 genes in RMA and dChip.

We compared the annotations of each method and of the combined one using the Fisher exact test [30] for the list of differentially expressed genes against the remaining genes in the array. We found that the combined method enriches 7 additional biological processes compared to those found by RMA, 5 additional biological processes and 3 additional cellular components compared to those found by MAS5 and 3 additional biological processes compared to those found by dChip.

The most represented KEGG pathways are related to immune response and oxidation (*Antigen processing and presentation*, *MAPK signalling pathway*, *PPAR signaling pathway*), biological regulators of physiological functions as energy metabolism, insulin action, immunity and inflammation and known from the literature to be associated with high fat diet (see [31] and [32]). Out of the KEGG pathways only the *Insulin signaling pathway *is enriched for the combined method with respect to the annotations of each single method.

## Discussion

The Bayesian calibration model that we propose makes use of the dependence between the results of the pre-processing methods and in doing so it gives a better assessment of the 'true' unknown differential expression between the conditions. Note that our calibration model relies on having generally applicable pre-processing methods for single arrays, and it is not aimed at replacing any of these as its applicability is limited to differential expression assessment, but it demonstrates that when dealing with several pre-processing methods, there is a good alternative to choosing just one for assessing differential expression. By using natural assumptions on the structure of the measurement errors it enables (i) to borrow information across the genes to estimate the method specific operational characteristics (*λ*_*j*_, *ϕ*_*j*_) and (ii) to borrow information across the methods to estimate a measure of differential expression (*δ*_*g*_) and a component of variability $\left(\sigma_{g}^{2}\right)$ specific to each probeset.

The reader should bear in mind that for the sake of clarity, the simulation has been set to represent a wide range of pre-processing methods, thus large differences between the parameters *λ*_*j *_and *ϕ*_*j *_for the 5 methods included were set. Note that when the bias is larger than 1, the signal is inflated, hence conditions are more favorable to detect differential expression. This does not mean that, paradoxically, the best pre-processing method is always characterised by the largest bias as the variability plays also a key role. The method that shows the best performance in terms of sensitivity and specificity achieves a balance between degree of inflation and variability. A realistic scenario is presented for the Latin Square dataset in Table 2 and 3 where RMA shows the best performance and is characterised by a relative bias *ϕ*_*j *_= 1.14 larger than dChip, but smaller than MAS5.

The inflation/deflation of differential expression found by the different methods could be related to the different steps that build each pre-processing method. MAS5 is the only method that normalises each array separately, while the internal normalisation of RMA and dChip forces the distribution to be the same between arrays and between conditions. This tends to reduce the width of the distribution, and can impact on the differential expression, leading to some shrinkage. Moreover, it has been shown in the literature that methods that subtract the mismatch are subject to higher variability in the expression ([33] and [34]). This finds confirmation in our examples as MAS5 is the only method included in the model that considers the mismatch correction and it exhibits the largest variability within biological replicates.

We want to stress the importance of having a way to assess whether the formulation of the synthetic model is an adequate representation of what is common and specific to the different methods. Bayesian model checks based on prediction allow the comparison of the observed data and the 'predicted' data under the model with respect to any feature of interest. We believe that the Mixed Posterior Predictive checks that we considered are effective for model criticism. They have the advantage of calculating a measure of discrepancy (the empirical p-value) for each gene that can be easily displayed through a histogram. On the Latin square and the experimental data we chose a flexible and realistic model, that allows a relation between the expression $\left({\bar{y}}_{g}\right)$ and the variance $\sigma_{gk}^{2}$. Comparing this formulation to a simpler version which assumes no relation between variability and level of expression by means of the Mixed Posterior Predictive checks, we clearly see a better fit for the more flexible model, which indicates that a variance parametrisation linked to the level of expression is better suited to the complexity of experimental data.

We have also calculated the Deviation Information Criteria (DIC) for each model and reached the same conclusions (see Tables 4, 5 in Additional file 1). We recommend that researchers use these tools to check the fit of the model to the data in hand and to compare different formulation. If necessary possible model extensions that might be appropriate to particular cases will need to be formulated and investigated. As the model proposed is designed for differential expression studies it seems reasonable that the bias parameter is proportional to the differential expression index *δ*_*g*_, but independent of the expression level *α*_*gj*_. The level of expression is taken into account through the model of the variance *σ*_*gjk *_for each pre-processing method. Nevertheless, on the Latin Square dataset we investigated the performance of a model where the bias is a polynomial function of the gene expression level (*ϕ*_1*j *_+ $\phi_{2j}{\bar{y}}_{g}$). It shows a worse fit than the model with only one bias parameter *ϕ*_*j*_, when considering both Mixed Posterior Predictive checks and the Deviance Information Criteria. Figure 6 of Additional file 1 shows the histogram for the Mixed Posterior Predictive check and Table 4 of Additional file 1 presents the Deviance Information Criteria for the extended model.

**Table 5:** Performance of simulated data on *ϕ *and *λ*

	*E*(*λ*_*j *_| **y**) [*CI*95%]	*E*(*ϕ*_*j *_| **y**) [*CI*95%]	sd (*λ*_*j*_)	sd (*ϕ*_*j*_)
Method 1 (*λ *= 1, *ϕ *= 1)	1.0 [1.0-1.0]	1.0 [1.0-1.0]	n.a.	n.a.
Method 2 (*λ *= 0.5, *ϕ *= 0.5)	0.5 [0.49–0.51]	0.5 [0.49–0.51]	0.03	0.04
Method 3 (*λ *= 0.5, *ϕ *= 2)	0.50 [0.49–0.51]	2.0 [1.97–2.04]	0.04	0.05
Method 4 (*λ *= 2, *ϕ *= 0.5)	2.0 [1.96–2.07]	0.5 [0.48–0.51]	0.06	0.04
Method 5 (*λ *= 2, *ϕ *= 2)	2.0 [1.97–2.04]	2 [1.99–2.03]	0.05	0.03

The table reports the posterior mean with the 95% credibility interval for the measurement error parameters, together with the true values set in the simulation. For all the parameters, the posterior mean coincides with the true value and is characterised by a small variability around it. The values are averaged over 10 runs. The empirical standard deviation over the 10 runs is also reported in the table. Note that the stardard deviation is not available for Method 1 as it is assumed to be the reference (*λ *= 1, *ϕ *= 1).

As already pointed out in the Results section, we have carried out an empirical investigation of the relative performance of the multiplicative bias model presented in (1) and of the additive bias model presented in (2). The pairwise comparison of the differential expression for the *J *methods considered supports the multiplicative bias formulation (Figure 1 and 2 of Additional file 1). In addition we reported the Deviance Information Criteria (DIC) for the Latin Square dataset in Table 4 and for the High Fat Diet dataset in Table 5 of Additional file 1. For both experiments, the DIC confirms the worst fit of the additive bias model.

We showed that combining several pre-processing methods is a way of including more information about the differential expression and that this improves the performance against each single method. We illustrated this using three commonly used pre-processing methods for Affymetrix chips, but we want to point out that our approach is generic in nature and that other pre-processing could be added or substituted instead of the three considered, as may be deemed appropriate by the analyst. We have carried out a limited investigation where we found that (i) including gcRMA [33] instead of RMA improves slightly the performance of the combining method, and (ii) including the vsn method [35] as expected alters the estimated parameters for the variability as vsn corrects for the dependence between the expression level and the variability, so the posterior estimates of *λ*_2*jk *_and *λ*_3*jk *_are very close to 0.

### Further extensions

The model proposed is designed for studies where the primary interest is the estimate of differential expression. Even if this is not the focus of the present paper, we want to point out that our methodology could be adapted to deal with studies where the interest is the quantification of the signal. Depending on the pre-processing methods, calibration models for quantifying the gene expression level would include multiplicative or additive bias, which might be a certain functions of the expression level. Our approach is also applicable to experiments where more than two conditions are included (see Bochkina and Richardson [24] for the methodology related to Bayesian hierarchical models for multiclass studies). Other extensions could be of interest: instead of returning a measure of differential expression for each probeset, the model could be modified to obtain a measure for each gene, adding a new component for estimating the variability between probesets mapping on the same gene. Moreover, the approach could easily be adapted to deal with data coming from different types of chips (e.g. Agilent, Illumina).

## Conclusion

This paper presents a Bayesian calibration model to synthesise information from several pre-processing methods. It is framed in a measurement error perspective, where each method is assumed to produce a biased measure of the latent true variable of interest. We specified the model for Affymetrix chips and focussed attention on differential expression studies, but we pointed out that the model is a methodological contribution and that our approach is applicable to a wide range of data types and experimental contrasts.

## Methods

### Simulated data

To test the performance of our method we simulated log expression values for 1000 genes, two conditions and five pre-processing methods from the model previously described, and extracted 5 replicates for each combination of condition and pre-processing (*r *= 1, ..., 5). We specified 200 differentially expressed genes characterised by a log expression $y_{gjkr}\sim N(\alpha_{gj}+{\left(-1\right)}^{k}\frac{1}{2}\delta_{g}\times \phi_{j},\sigma_{gj}^{2})$, with *k *= 1, 2, while for the remaining 800 genes the log expressions for both conditions came from the same distribution: $y_{gjkr}\sim N(\alpha_{gj},\sigma_{gj}^{2})$. The parameters for simulating the distributions of *α*_*gj *_~*N *(6.79, 4.77) and *δ*_*g *_~*N *(0, 0.25) were obtained from experimental data we have analysed [36]; the model on the variance $\sigma_{gjk}^{2}$ is presented in equation (3) with (*λ*_2*jk *_= 0, *λ*_3*jk *_= 0) and we assume the same variance for the two conditions $\left(\sigma_{gj1}^{2}\equiv \sigma_{gj2}^{2}\right)$, obtained from experimental data, where the mean is 0.03 and the distribution can be summarised by the following quartiles: 0.02, 0.04, 0.09 and 0.15.

The 5 pre-processing methods are described in table 7:

**Table 7:** Values of the parameters for the 5 pre-processing methods in the simulation scenario

	Method 1	Method 2	Method 3	Method 4	Method 5
*exp*(*λ*_*j*_)	1	0.5	0.5	2	2
*ϕ*_*j*_	1	0.5	2	0.5	2

We assume that the first method is the 'reference', being characterised by *exp*(*λ*_1_) = 1 and *ϕ*_1 _= 1. Method 2 and Method 4 have a smaller relative bias (*ϕ*_2 _= *ϕ*_4 _= 0.5), while that of Method 3 and Method 5 is larger (*ϕ*_3 _= *ϕ*_5 _= 2); Method 2 and Method 3 have a variability smaller than 1 (*exp*(*λ*_2_) = *exp*(*λ*_3_) = 0.5) while that of Method 4 and Method 5 is larger (*exp*(*λ*_4_) = *exp*(*λ*_5_) = 2).

We used an MCMC algorithm with two chains to estimate the parameters of interest (we checked convergence for 10000 iterations and then extracted a sample of 1000 iterations so that the MC error was smaller that the 5% of the sample standard deviation, as recommended).

To evaluate the consistency of our results we repeated the simulation process 10 times and performed our Bayesian analysis for each run. The model estimates well the values of the parameters *λ *and *ϕ *(see Table 5 for their posterior mean and 95% credibility intervals; see Figures 7 and 8 of Additional file 1 for the associated posterior density plots).

As a point of comparison we also ran the model separately for each pre-processing method and compared the performance of both combined and single pre-processing methods in terms of sensitivity and specificity (see the results on simulated data).

### Spike-in example: Latin Square Affymetrix data

We tested our method by means of the Affymetrix Latin Square data set [21]. The array used is human *Hgu*133*a *and contains 22300 probesets. There are 14 experimental conditions and each has 3 replicates. We considered the experimental condition 2 versus 1 and the 42 spike-in probesets indicated by Affymetrix plus the 22 new spike-in probesets proposed by McGee *et al*. [37]. The ratio of the concentrations is 2:1 for 60 spike-in probesets and 0 for the remaining 4. Among the remaining 22236 probesets we extracted only the ones present in at least one condition, evaluated using the present/absent call included in the Affy R package  and obtained 10621 probesets.

We focused attention on the three most used pre-processing methods : MAS5 [38], RMA [39] and dChip [40]. There are many versions of each of them, but we considered the default ones for MAS5 and RMA and the one obtained by the expresso function for dChip. All are provided by the Bioconductor project [41]. The differences in the three methods are described in Table 6.

**Table 6:** Characteristics of MAS5, RMA and dChip

	Background correction	Perfect Match correction	Normalisation	Summary
MAS5	Divide the chip in 16 regions. The lowest 2% is the background. Weighted average over all the regions.	Ideal Mismatch	Scaling	1 step Tukey Biweight
RMA	Global model for the distribution of the probe intensities	No correction	Quantile	Median Polish
dChip	No correction	No correction	Invariant set using one array as default	Multi-chip linear model

Characteristics of MAS5, RMA and dChip in terms of perfect match correction, normalization and summarization method.

We ran the combined model, but also treated separately each pre-processing method. Again we performed the MCMC estimation with two chains (we checked convergence for 10000 iterations and then extracted a sample of 1000 iterations, characterised by a MC error smaller than 5% of the sample standard deviation, as recommended). The MCMC simulation of the combined model takes 9 hours and 52 minutes to run 11000 iterations on the 10621 probesets.

To see if the results are consistent we ran the model on additional pairwise comparisons, selected to represent a wide range of real differential expression (real fold change ranging from 0.002 to 128). The behavior is similar for the variability parameters *λ *across the experiments and the relative bias *ϕ *shows the same ranking for all the comparisons (MAS5>RMA>dChip). Also the performance of the methods is consistent in all the comparisons with the combined method showing the highest specificity and sensitivity. The results are presented in Tables 1, 2, 3 of Additional file 1.

### Biological example: High Fat Diet versus Normal Fat Diet in mice adipose tissue

There are many studies in the literature describing the effect of high fat diet on gene expression of several tissues in mice (see for example [42] and [43] for adipose tissue and [44] for liver). They are particularly interesting since the effect of diet can trigger obesity, hypertension and be related to major pathologies as diabetes. In order to assess if our model leads to a more powerful analysis that improves the biological interpretability of the results we ran it on a publicly available experiment to study the effect of high fat diet (HFD) versus normal fat diet (NFD) on mice adipose tissue. The array used is mouse *MG*_*U*74*Av*2 and contains 12488 probesets. The experiment analyzes the strain 129 and for each condition there are 4 replicates. The .CEL files and the description of the experiments are available at the DGAP project website [29].

Again we pre-processed the data using MAS5, RMA and dChip and ran the combined model, but also treated separately each pre-processing method. The MCMC estimation was performed with two chains (we checked convergence for 10000 iterations and then extracted a sample of 1000 iterations. The MC error is smaller than the 5% of the sample standard deviation as recommended). The MCMC simulation for the combined model takes 12 hours and 10 minutes to run 11000 iterations on the 12488 probesets.

### Implementation

The standard model built by equations (1), (3), (4) and by the prior distributions has been implemented in the free software WinBUGS and the code is provided in Additional file 1. All the analyses were performed on a DELL Precision workstation with 3.20 GHz and 2 GB of RAM. Note that it is relatively quick to run with a small number of genes (it takes around 5 minutes to perform 1000 iterations for 1000 genes, 2 conditions, 3 pre-processing and 5 replicates), but the time increases linearly with the number of genes. To increase the speed on the Latin Square data set (that includes 22300 probesets), we filtered the present probesets for at least one condition, using the present/absent call implemented in the Affy R package; this halved the computational time (from 20 to around 10 hours for 11000 iterations). If needed it is possible to apply different or more stringent criteria (e.g. selecting the most variable genes between the two conditions), as long as the non differentially expressed genes are well represented in the subset, otherwise the null distribution of *δ*_*g*_, and in particular its variability, is not properly identified and it can affect the correct estimate of the tail posterior probability.

## Authors' contributions

All authors conceived the model and the structure of the paper. MB performed the analysis and drafted the paper. All authors read and approved the final manuscript.

## Supplementary Material

Additional file 1 **Supplementary Information**. It provides supplementary information about the methodology used, including additional plots, tables and the WinBUGS code for the calibration model. Click here for file
