# Supplementary material for: A Bayesian calibration model for combining different pre-processing methods in Affymetrix chips
Source: BMC Bioinformatics. 2008 Dec 1;9:512. doi: 10.1186/1471-2105-9-512 (PMC2639433; doi:10.1186/1471-2105-9-512)
Supplement: Additional file 1 — Supplementary Information. It provides supplementary information about the methodology used, including additional plots, tables and the WinBUGS code for the calibration model. [file 1471-2105-9-512-S1.pdf]

Supplementary Information

Marta Blangiardo*[1]and Sylvia Richardson[1]

[1]Centre for Biostatistics, Imperial College. St. Mary's Campus, Norfolk Place London W2 1PG, UK

Email: MB: m.blangiardo@imperial.ac.uk; SR: sylvia.richardson@imperial ac.uk;

*Corresponding author

1 Posterior Predictive Check

The Posterior Predictive Check compares the observed sample variance, calculated on the expression values, and the variance of the predicted expression values of each gene under the model using an empirical p-value.
The strategy for calculating the posterior predictive check can be described as follows:

1. A new value for the gene specific component of the variance for each condition ($\sigma_{gk}^{2(pred)}$) is predicted from its prior distribution $Ga^{-1}(a_k, b_k)$
2. new data y_{gjkr}^{pred} are generated under the chosen model, current values of the parameters α_{gj}, δ_g, λ_j, ϕ_j and the predicted variance:

$$y_{gjkr}^{pred} \sim N(\alpha_{gj} + (-1)^k \frac{1}{2} \times \delta_g \times \phi_j, \sigma_{gjk}^{2(pred)}) \qquad k = 1, 2.$$

   where the variability has the same structure presented in (2),

$$\sigma_{gjk}^{2(pred)} = \exp(\lambda_{1jk} + \lambda_{2jk} \times \overline{y}_g + \lambda_{3jk} \times \overline{y}_g^2) \times \sigma_{gk}^{2(pred)}.$$

3. For each gene and pre-processing method the predicted sample variance ($S_{gjk}^{2(pred)} = \frac{1}{2R-1} \sum_{r=1}^{2R} (y_{gjkr}^{(pred)} - \overline{y}_{gjk}^{(pred)})^2$) is compared to the observed sample variance ($S_{gjk}^{2(obs)} = \frac{1}{2R-1} \sum_{r=1}^{2R} (y_{gjkr} - \overline{y}_{gjk})^2$) through the statistic ($S_{gjk}^{2(pred)} - S_{gjk}^{2(obs)}$) and a p-value is generated.

Under the null hypothesis of the model being true, the distribution of the p-values should be approximatively uniform, while a poor model fit is indicated by the presence of a notable pattern in the plot, suggesting a systematic difference between the observed values and the predicted ones.

2 Tables and Figures

	Exp 2 vs 4		Exp 3 vs 6		Exp 4 vs 8	
	$E(\lambda\|y)$	CI95%	$E(\lambda\|y)$	CI95%	$E(\lambda\|y)$	CI95%
λ_{111}	-7.005	-7.069, -6.965	-6.718	-6.779, -6.674	-7.451	-7.490, -7.417
λ_{121}	4.658	4.599, 4.712	4.478	4.429, 4.521	5.642	5.597, 5.696
λ_{131}	2.347	2.299, 2.378	2.239	2.182, 2.294	1.809	1.766, 1.848
λ_{211}	1.578	1.561, 1.583	1.395	1.381, 1.409	1.558	1.551, 1.565
λ_{221}	-1.274	-1.281, -1.259	-1.214	-1.219, -1.207	-1.540	-1.555, -1.528
λ_{231}	-0.303	-0.309, -0.296	-0.181	-0.197, -0.163	-0.018	-0.029, -0.008
λ_{311}	-0.097	-0.098, -0.096	-0.083	-0.084, -0.081	-0.090	-0.091, -0.089
λ_{321}	0.082	0.081, 0.082	0.080	0.079, 0.081	0.103	0.102, 0.104
λ_{331}	0.016	0.015, 0.016	0.002	0.001, 0.004	-0.013	-0.014, -0.012
λ_{112}	-5.746	-5.780, -5.706	-6.580	-6.626, -6.536	-6.803	-6.886, -6.721
λ_{122}	3.726	3.682, 3.768	4.535	4.489, 4.609	5.134	5.087, 5.183
λ_{132}	2.020	1.968, 2.064	2.045	1.993, 2.091	1.669	1.623, 1.715
λ_{212}	1.058	1.050, 1.063	1.466	1.453, 1.483	1.422	1.408, 1.439
λ_{222}	-0.942	-0.949, -0.932	-1.249	-1.275, -1.230	-1.409	-1.415, -1.402
λ_{232}	-0.116	-0.128, -0.108	-0.216	-0.225, -0.208	-0.013	-0.024, -0.002
λ_{312}	-0.055	-0.056, -0.055	-0.088	-0.089, -0.086	-0.084	-0.084, -0.082
λ_{322}	0.058	0.057, 0.059	0.079	0.077, 0.081	0.091	0.090, 0.091
λ_{332}	-0.003	-0.004, -0.002	0.009	0.008, 0.009	-0.007	-0.008, -0.006
	Exp 5 vs 10		Exp 6 vs 12		Exp 7 vs 14	
	$E(\lambda\|y)$	CI95%	$E(\lambda\|y)$	CI95%	$E(\lambda\|y)$	CI95%
λ_{111}	-7.048	-7.092, -6.987	-6.599	-6.643, -6.558	-6.904	-6.960, -6.831
λ_{121}	4.109	3.994, 4.199	3.913	3.841, 4.006	4.387	4.330, 4.437
λ_{131}	2.939	2.879, 3.012	2.686	2.637, 2.742	2.516	2.484, 2.540
λ_{211}	1.585	1.565, 1.603	1.332	1.322, 1.351	1.490	1.476, 1.506
λ_{221}	-1.185	-1.222, -1.150	-1.058	-1.077, -1.036	-1.184	-1.197, -1.171
λ_{231}	-0.400	-0.417, -0.381	-0.275	-0.287, -0.267	-0.306	-0.314, -0.300
λ_{311}	-0.100	-0.103, -0.098	-0.076	-0.078, -0.075	-0.089	-0.090, -0.087
λ_{321}	0.081	0.078, 0.085	0.068	0.067, 0.070	0.076	0.075, 0.077
λ_{331}	0.019	0.017, 0.021	0.007	0.006, 0.009	0.013	0.012, 0.014
λ_{112}	-7.832	-7.876, -7.792	-6.248	-6.291, -6.189	-6.994	-7.047, -6.940
λ_{122}	4.581	4.539, 4.618	3.939	3.89, 3.983	4.491	4.436, 4.545
λ_{132}	3.252	3.207, 3.279	2.309	2.269, 2.344	2.504	2.463, 2.547
λ_{212}	1.740	1.730, 1.747	1.368	1.359, 1.377	1.553	1.540, 1.570
λ_{222}	-1.278	-1.287, -1.268	-1.077	-1.094, -1.068	-1.224	-1.236, -1.209
λ_{232}	-0.462	-0.472, -0.450	-0.290	-0.297, -0.281	-0.329	-0.340s, -0.318
λ_{312}	-0.110	-0.110, -0.109	-0.080	-0.081, -0.079	-0.094	-0.096, -0.093
λ_{322}	0.087	0.086, 0.088	0.067	0.065, 0.069	0.078	0.077, 0.079
λ_{332}	0.023	0.022, 0.024	0.014	0.012, 0.015	0.016	0.014, 0.018

Table 1: Latin Square data set: Posterior Mean and 95% credibility intervals for λ_1, λ_2 and λ_3 in the variability model presented in (2) for additional comparisons to check the consistence of the results. $j = 1, 2, 3$ indicates the pre-processing method, $k = 1, 2$ indicates the condition. The coefficients satisfy the "sum to 0" constraint.

	Exp 2 vs 4		Exp 3 vs 6		Exp 4 vs 8	
	$E(\phi\|y)$	CI95%	$E(\phi\|y)$	CI95%	$E(\phi\|y)$	CI95%
MAS5	1.452	1.438, 1.464	1.530	1.516, 1.554	1.216	1.207, 1.223
RMA	1.049	1.041, 1.059	1.063	1.044, 1.076	1.236	1.228, 1.245
dChip	0.656	0.651, 0.660	0.615	0.606, 0.622	0.666	0.662, 0.669
	Exp 5 vs 10		Exp 6 vs 12		Exp 7 vs 14	
	$E(\phi\|y)$	CI95%	$E(\phi\|y)$	CI95%	$E(\phi\|y)$	CI95%
MAS5	1.283	1.276, 1.292	1.460	1.451, 1.469	1.297	1.297, 1.326
RMA	1.185	1.178, 1.192	1.098	1.091, 1.103	1.140	1.132, 1.148
dChip	0.658	0.656, 0.660	0.624	0.620, 0.627	0.679	0.670, 0.687

Table 2: Latin Square data set: Posterior Mean and 95% credibility intervals for the relative bias $\exp(\phi_j)$ for additional comparisons to check the consistence of the results. $j = 1, 2, 3$ indicates the pre-processing method. The coefficients satisfy the constraint that $\prod \phi_j = 1$.

	Exp 2 vs 4				Exp 3 vs 6			
	First 64 ranked genes				First 64 ranked genes			
	FP	TN	TP	FN	FP	TN	TP	FN
Combined	13	11331	51	13	12	11334	52	12
MAS5	18	11326	46	18	18	11328	46	18
RMA	16	11328	48	16	15	11331	49	15
dChip	21	11323	43	21	34	11312	30	34
	Exp 4 vs 8				Exp 5 vs 10			
	First 64 ranked genes				First 64 ranked genes			
	FP	TN	TP	FN	FP	TN	TP	FN
Combined	20	11233	44	20	18	11448	46	18
MAS5	29	11224	35	29	23	11443	41	23
RMA	23	11230	41	23	20	11446	44	20
dChip	34	11219	30	34	28	11438	36	28
	Exp 6 vs 12				Exp 7 vs 14			
	First 64 ranked genes				First 64 ranked genes			
	FP	TN	TP	FN	FP	TN	TP	FN
Combined	9	11305	55	9	13	11368	51	13
MAS5	15	11299	49	15	26	11355	38	26
RMA	11	11303	53	11	19	11362	45	19
dChip	19	11295	45	19	33	11348	31	33

Table 3: Latin Square dataset: operating characteristics of the combined method and of each pre-processing method on the first 64 probesets ranked accordingly to their tail posterior probability for additional comparisons to check the consistence of the results. Note that $FP = FN$ since the size of the list of differentially expressed probesets is equal to the number of *true positives.* The times for running the combined model for each comparison are the following: 10h and 40', 10h and 41', 10h and 28', 10h and 53', 10h and 37', 10h and 46'.

Model specification		
Variability	Bias	DIC
λ_{jk}	multiplicative bias function of global expression ($\phi_{j1} + \phi_{j2}\overline{y}_g$)	-452010
$\lambda_{1jk} + \lambda_{2jk}\overline{y}_g + \lambda_{3jk}\overline{y}_g^2$	multiplicative bias function of global expression ($\phi_{j1} + \phi_{j2}\overline{y}_g$)	-463560
λ_{jk}	multiplicative bias (ϕ_{j1})	-462127
$\lambda_{1jk} + \lambda_{2jk}\overline{y}_g + \lambda_{3jk}\overline{y}_g^2$	multiplicative bias (ϕ_{j1})	-473836
$\lambda_{1jk} + \lambda_{2jk}\overline{y}_g + \lambda_{3jk}\overline{y}_g^2$	additive bias (ξ_{j1})	-453210

Table 4: Deviation Information Criteria for different models on the Latin Square dataset

Model specification		
Variability	Bias	DIC
λ_{jk}	multiplicative bias (ϕ_{j1})	92331
$\lambda_{1jk} + \lambda_{2jk}\overline{y}_g + \lambda_{3jk}\overline{y}_g^2$	multiplicative bias (ϕ_{j1})	63964
$\lambda_{1jk} + \lambda_{2jk}\overline{y}_g + \lambda_{3jk}\overline{y}_g^2$	additive bias (ξ_{j1})	67795

Table 5: Deviation Information Criteria for different models on the High Fat Diet dataset

	$E(\lambda \mid \mathbf{y})$	$CI95\%$
$\lambda_{1,j=1,k=1}$	-5.96	-6.14 , -5.25
$\lambda_{1,j=2,k=1}$	4.30	4.17 , 4.44
$\lambda_{1,j=3,k=1}$	1.66	1.50 , 1.78
$\lambda_{2,j=1,k=1}$	1.22	1.19 , 1.27
$\lambda_{2,j=2,k=1}$	-1.14	-1.18 , -1.10
$\lambda_{2,j=3,k=1}$	-0.08	-0.11 , -0.03
$\lambda_{3,j=1,k=1}$	-0.07	-0.07 , -0.07
$\lambda_{3,j=2,k=1}$	0.07	0.07 , 0.08
$\lambda_{3,j=3,k=1}$	-0.00	-0.01 , 0.01
$\lambda_{1,j=1,k=2}$	-7.07	-7.20 , -6.94
$\lambda_{1,j=2,k=2}$	4.82	4.66 , 4.93
$\lambda_{1,j=3,k=2}$	2.25	2.11 , 2.36
$\lambda_{2,j=1,k=2}$	1.61	1.58 , 1.65
$\lambda_{2,j=2,k=2}$	-1.36	-1.39 , -1.31
$\lambda_{2,j=3,k=2}$	-0.25	-0.28 , -0.21
$\lambda_{3,j=1,k=2}$	-0.10	-0.10 , -0.10
$\lambda_{3,j=2,k=2}$	0.09	0.09 , 0.09
$\lambda_{3,j=3,k=2}$	0.01	0.01 , 0.01

Table 6: Latin Square data set: Posterior Mean and 95% credibility intervals for λ_1, λ_2 and λ_3 in the variability model presented in (2). $j = 1, 2, 3$ indicates the pre-processing method, $k = 1, 2$ indicates the condition. The coefficients satisfy the “sum to 0” constraint.

	$E(\lambda \mid \mathbf{y})$	$CI95\%$
$\lambda_{1,j=1,k=1}$	-4.791	-4.811 , -4.771
$\lambda_{1,j=2,k=1}$	2.550	2.522 , 2.575
$\lambda_{1,j=3,k=1}$	2.241	2.217 , 2.272
$\lambda_{2,j=1,k=1}$	0.743	0.736 , 0.749
$\lambda_{2,j=2,k=1}$	-0.590	-0.598 , -0.576
$\lambda_{2,j=3,k=1}$	-0.153	-0.161 , -0.146
$\lambda_{3,j=1,k=1}$	-0.024	-0.025 , -0.023
$\lambda_{3,j=2,k=1}$	0.028	0.027 , 0.029
$\lambda_{3,j=3,k=1}$	-0.004	-0.005 , -0.003
$\lambda_{1,j=1,k=2}$	-6.604	-6.638 , -6.575
$\lambda_{1,j=2,k=2}$	3.628	3.580 , 3.680
$\lambda_{1,j=3,k=2}$	2.976	2.948 , 3.001
$\lambda_{2,j=1,k=2}$	1.454	1.447 , 1.461
$\lambda_{2,j=2,k=2}$	-0.944	-0.957 , -0.933
$\lambda_{2,j=3,k=2}$	-0.510	-0.515 , -0.502
$\lambda_{3,j=1,k=2}$	-0.081	-0.082 , -0.081
$\lambda_{3,j=2,k=2}$	0.053	0.052 , 0.054
$\lambda_{3,j=3,k=2}$	0.028	0.028 , 0.029

Table 7: HFD vs NFD experiment: Posterior Mean and 95% credibility intervals for λ_1, λ_2 and λ_3 in the variability model presented in (2). $j = 1, 2, 3$ indicates the pre-processing method, $k = 1, 2$ indicates the condition. The coefficients satisfy the "sum to 0" constraint.

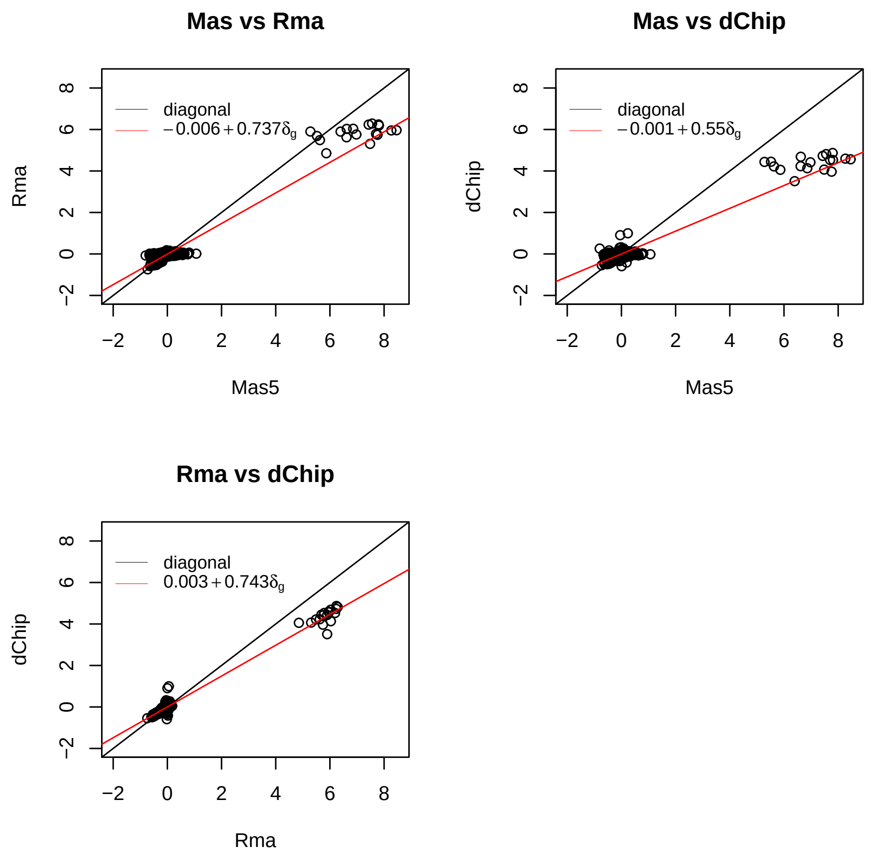

Figure 1: Latin Square dataset: the plots present the pairwise comparison of differential expression for the pre-processing methods considered. In absence of bias the data should lie on the diagonal (black line). If the additive component of the bias is the most relevant $(\alpha_{gj} + \delta_g + \xi_j)$, a shift should be visible in the differential expression of one pre-processing method with respect to the other. If the multiplicative component is more important $(\alpha_{gj} + \delta_g \times \phi_j)$ a twist should be present in the scatterplot, as one method is characterised by an inflation (or deflation) with respect to the other differential expression. Regressing the differential expression of one method to the other (red line), we see that the slope is different from 1 (representing the inflation/deflation in case of multiplicative bias), while the intercept is small and generally close to 0 (representing the shift in case of additive bias). This suggests that the bias is better represented by a multiplicative component of the differential expression.

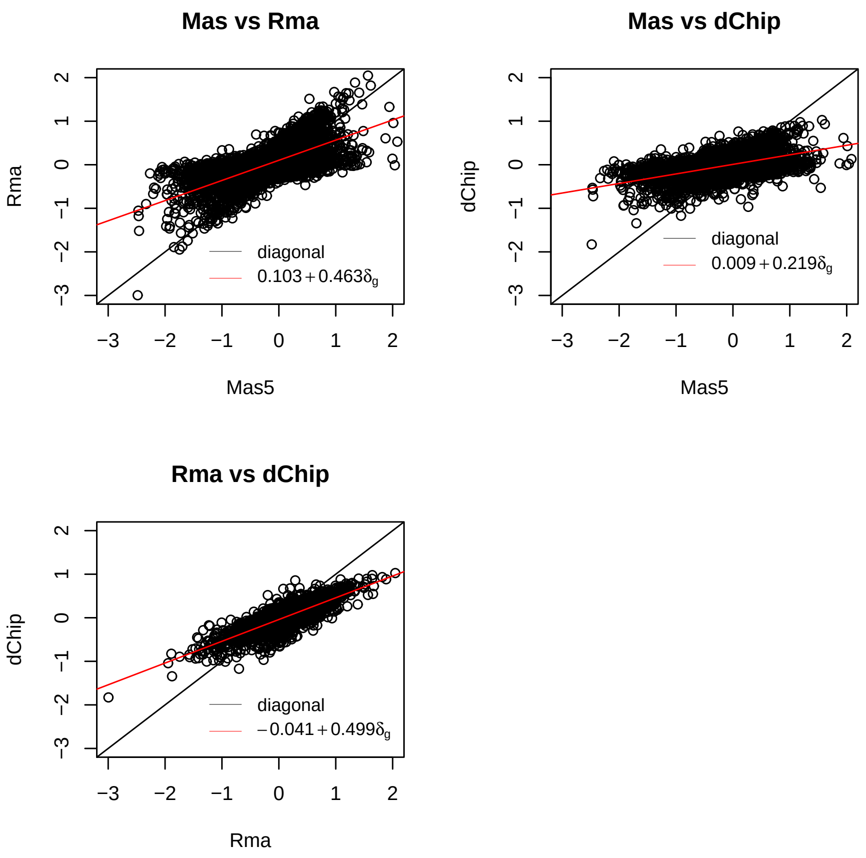

Figure 2: High Fat Diet vs Normal Fat Diet experiment: the plots present the pairwise comparison of differential expression for the pre-processing methods considered. In absence of bias the data should lie on the diagonal (black line). If the additive component of the bias is the most relevant ($\alpha_{gj} + \delta_g + \xi_j$), a shift should be visible in the differential expression of one pre-processing method with respect to the other. If the multiplicative component is more important ($\alpha_{gj} + \delta_g \times \phi_j$) a twist should be present in the scatterplot, as one method is characterised by an inflation (or deflation) with respect to the other differential expression. Regressing the differential expression of one method to the other (red line), we see that the slope is different from 1 (representing the inflation/deflation in case of multiplicative bias), while the intercept is small and generally close to 0 (representing the shift in case of additive bias). This suggests that the bias is better represented by a multiplicative component of the differential expression.

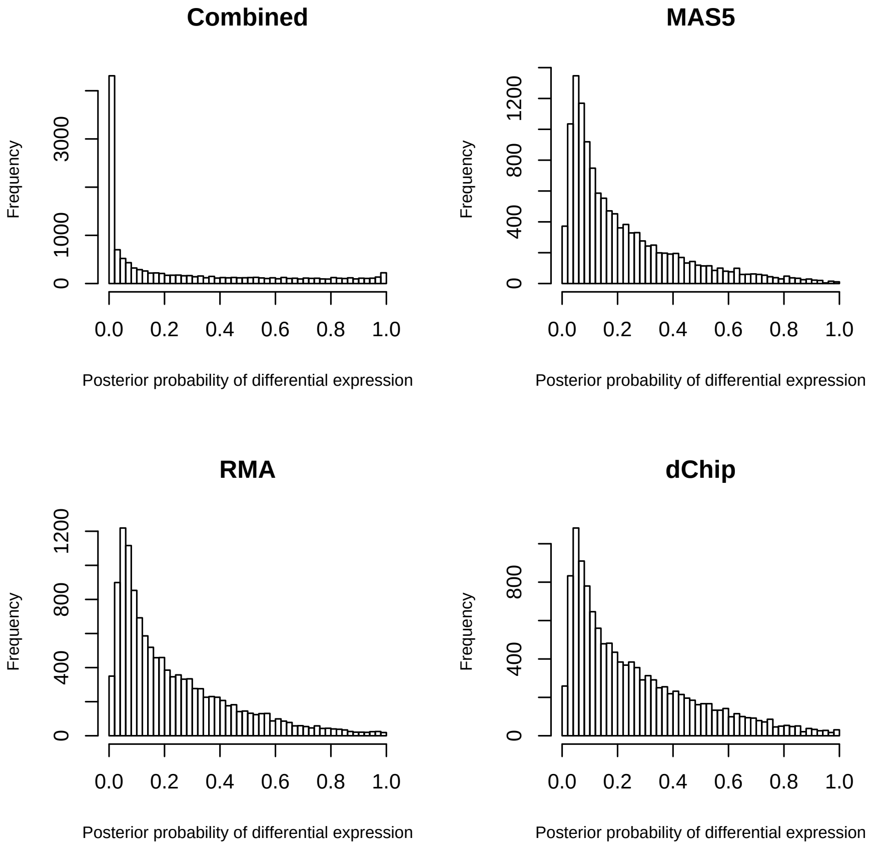

Figure 3: High Fat Diet vs Normal Fat Diet experiment: the histograms show the distribution of the tail posterior probability for the combined method and each pre-processing method separately, considering MAS5, RMA and dChip. In contrast to what happens for each single pre-processing method, the combined model shows a local peak on the right tail of the distribution, indicating evidence of differential expression.

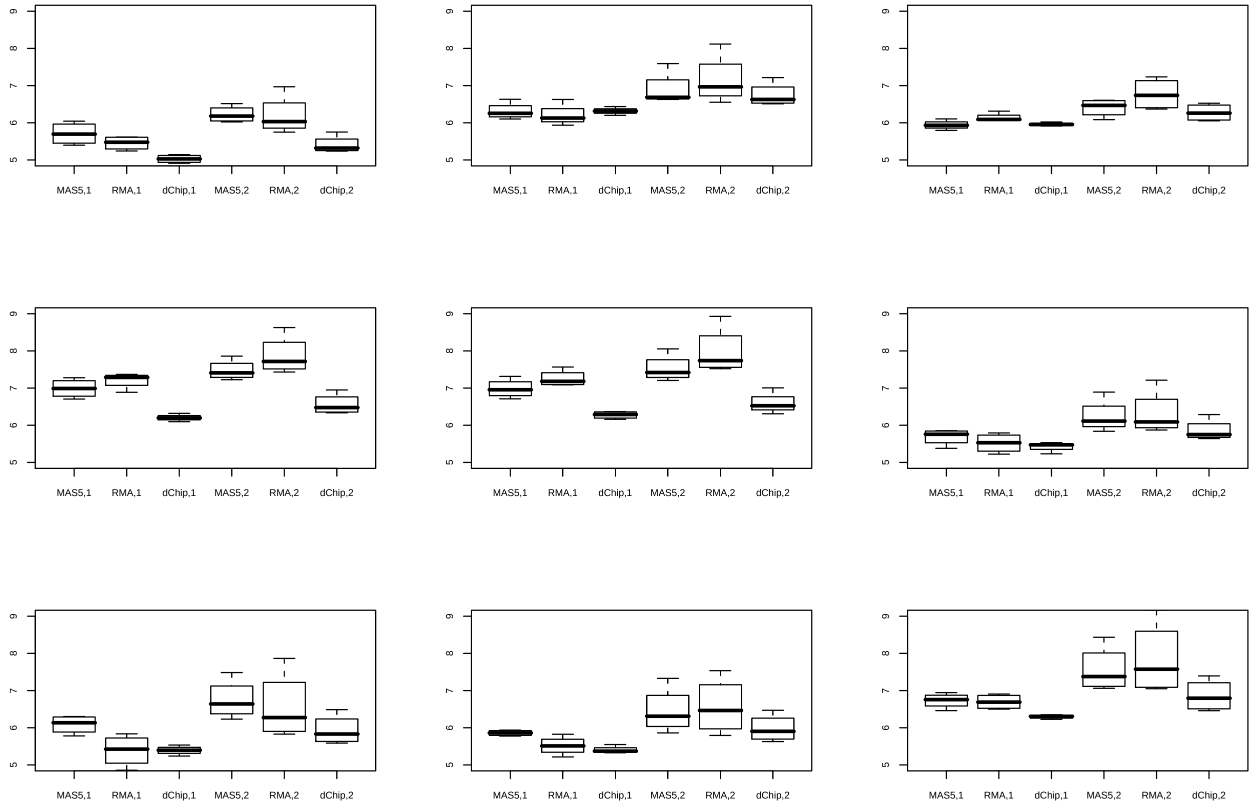

Figure 4: High Fat Diet vs Normal Fat Diet experiment: Box Plot for 9 out of the 61 probesets selected as differentially expressed only by the combined approach.

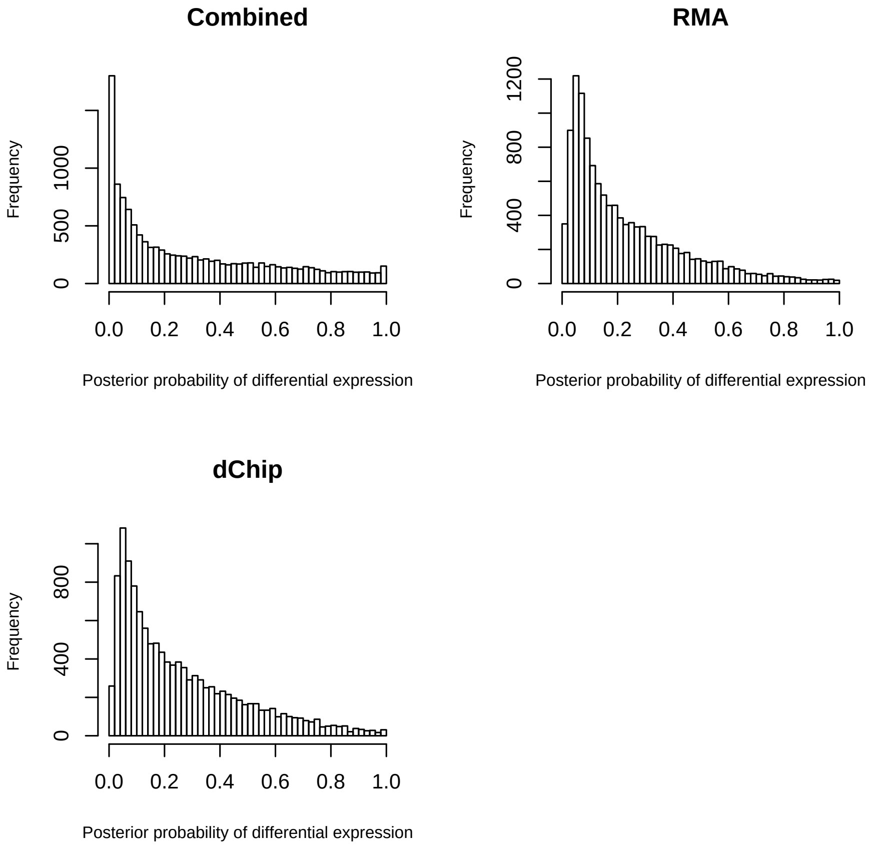

Figure 5: High Fat Diet vs Normal Fat Diet experiment: the histograms show the distribution of the tail posterior probability for the combined method and each pre-processing method separately, when we consider only RMA and dChip. In contrast to what happens for each single pre-processing method, the combined model shows a local peak on the right tail of the distribution, indicating evidence of differential expression.

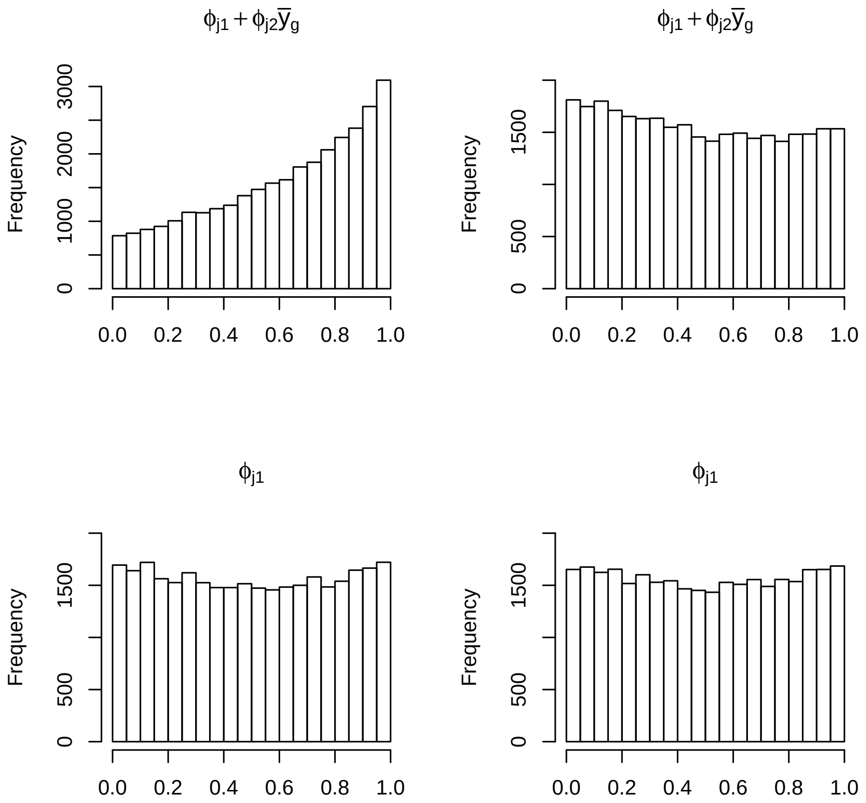

Figure 6: Posterior Predictive Check: comparing the models on the bias parameter. The upper plots are related to the model where the bias is a polynomial function of the level of expression. For the first condition is clearly visible a pattern deviating from the uniformity. The bottom plot is related to the model with only 1 bias for each pre-processing method. The p-values show a uniform behavior.

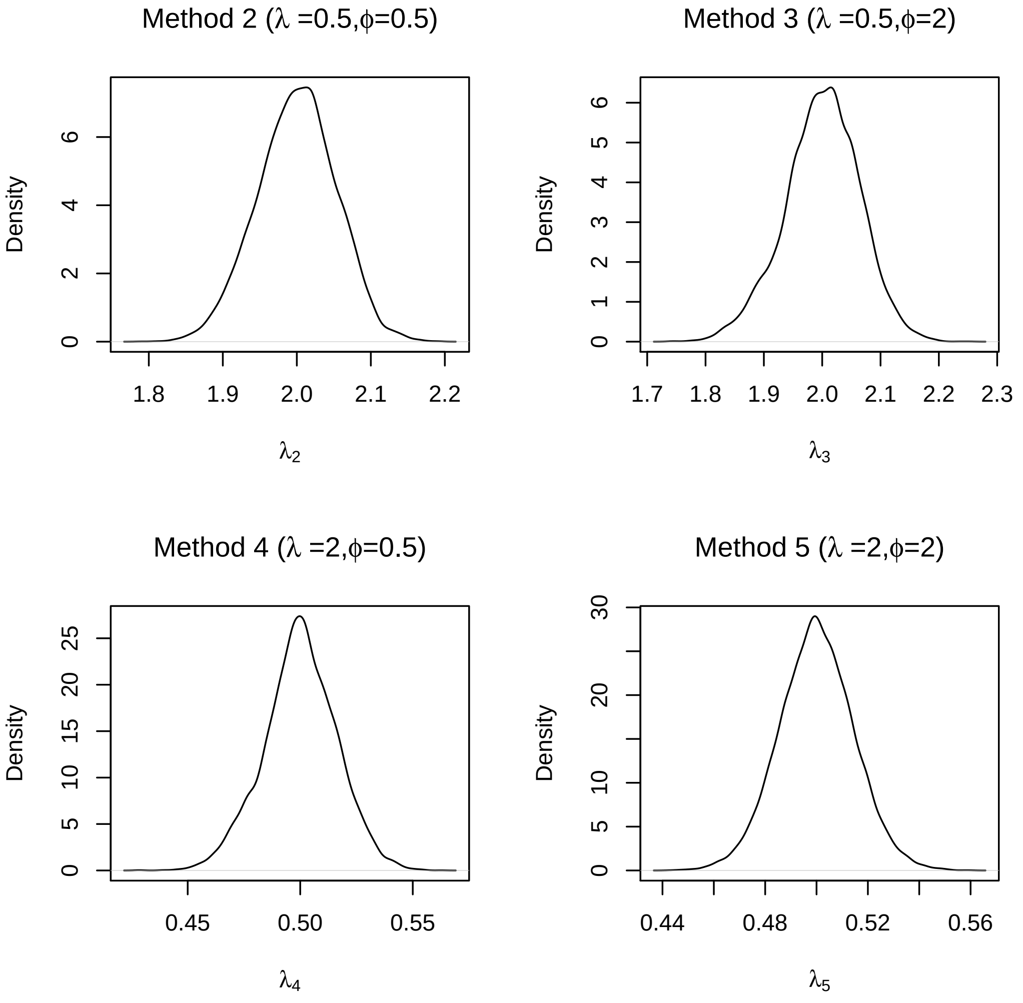

Figure 7: Density plots for λ_j $(j = 1, \ldots, 5)$. The Figure shows how in the simulation study the posterior λ_j distribution for each method is centered around the *true* value suggesting a good performance of the model in estimating the true error terms

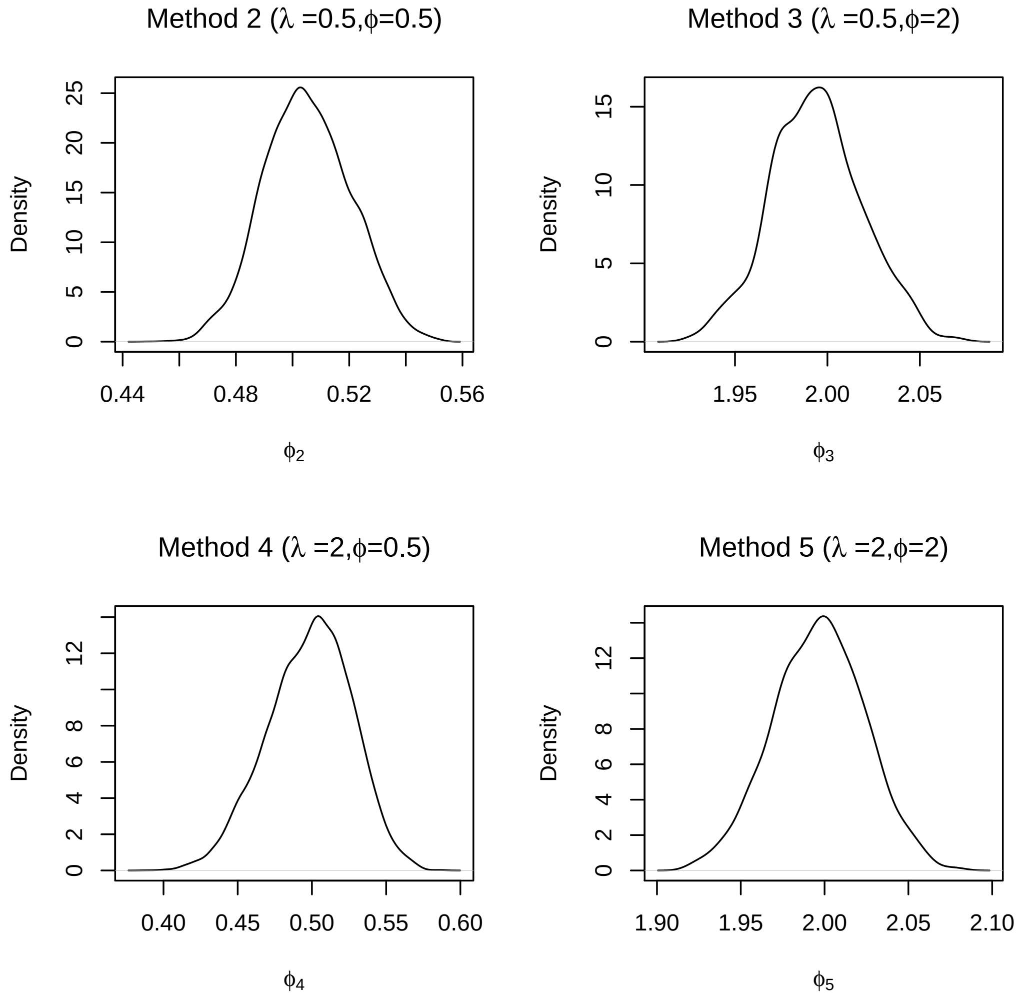

Figure 8: Density plots for ϕ_j ($j = 1, \ldots, 5$). The Figure shows how in the simulation study the posterior ϕ_j distribution for each method is centered around the *true* value suggesting a good performance of the model in estimating the true relative bias parameters

3 WinBUGS code

```
#Calibration model with the corner point constraint
#G=genes
#J=pre-processing
#K=replicates

model{

    for(g in 1:G){
        for(j in 1:J){
            for(k in 1:K){
            # log gene expression
            y1[g,j,k] ~ dnorm(mu[g,j,1],tau1[g,j])
            y2[g,j,k] ~ dnorm(mu[g,j,2],tau2[g,j])
            #Predicted values under the model
            y1new[g,j,k] ~ dnorm(mu[g,j,1],tau.new1[g,j])
            y2new[g,j,k] ~ dnorm(mu[g,j,2],tau.new2[g,j])

            }
        #ANOVA
        #Condition 1
        mu[g,j,1] <- alpha[g,j]  -  1/2*delta[g]*exp(d[j])
        #Condition 2
        mu[g,j,2] <- alpha[g,j] +  1/2*delta[g]*exp(d[j])


        #VARIANCE gene, method and condition specific
        tau1[g,j] <-exp(lambda1[1,j] + lambda1[2,j]*expr[g] + lambda1[3,j]*pow(expr[g],2))
        *tau.gene1[g]
        tau2[g,j] <-exp(lambda2[1,j] + lambda2[2,j]*expr[g] + lambda2[3,j]*pow(expr[g],2))
        *tau.gene2[g]
        tau.new1[g,j] <-exp(lambda1[1,j] + lambda1[2,j]*expr[g] + lambda1[3,j]*pow(expr[g],2))
        *tau.gene.new1[g]
        tau.new2[g,j] <-exp(lambda2[1,j] + lambda2[2,j]*expr[g] + lambda2[3,j]*pow(expr[g],2))
        *tau.gene.new2[g]

        #Global gene expression for each gene and method
        alpha[g,j] ~ dnorm(0,0.00001)
        }

    #Differential expression for each gene
```

```
    delta[g] ~ dnorm(0,0.0001)

    #Precisions and Variances for each gene and condition
    tau.gene1[g] ~ dgamma(a1,b1)
    sigma.gene1[g] <- 1/tau.gene1[g]

    tau.gene2[g] ~ dgamma(a2,b2)
    sigma.gene2[g] <- 1/tau.gene2[g]

    tau.gene.new1[g] ~ dgamma(a1,b1)
    sigma.gene.new1[g] <- 1/tau.gene.new1[g]

    tau.gene.new2[g] ~ dgamma(a2,b2)
    sigma.gene.new2[g] <- 1/tau.gene.new2[g]
    }

#Corner point constraint for the coefficients of the exponential
component of variability

    for(r in 1:R){
lambda1[r,1]<-0
lambda2[r,1]<-0
        for(j in 2:J){
            lambda1[r,j] ~dnorm(0.01,0.01)
            lambda2[r,j] ~dnorm(0.01,0.01)
            }
        }

#Corner point constraint for the relative bias coefficients

d[1]<-0
    for(j in 2:J){
    d[j] ~ dnorm(0.01,0.01)
    }

#Hyperparameters for the variance

a1~dgamma(0.01,0.01)
b1~dgamma(0.01,bstar1)

a2~dgamma(0.01,0.01)
```

```
b2~dgamma(0.01,bstar2)

bstar1 <- 1/pow(bdistr1,2)
bstar2 <- 1/pow(bdistr2,2)

bdistr1 ~ dunif(0,13.36)
bdistr2 ~ dunif(0,13.80)
}
```

4 Transforming corner point coefficients in sum to 0 coefficients

There are two types of constraints that can be used in the model: (i) corner point, (ii) sum to 0. Both constraints return the same results in terms of differential expression. We have applied the corner point that runs faster and then we have obtained the sum to 0 coefficients that are more easily interpretable. In this paragraph we provide the formula for extracting the "sum to 0" coefficients from the corner point ones. For $j = 1, \cdots, J$ methods we can specify the corner point constraint as follows:

$$\xi_j = \xi_1 + \beta_j \qquad (j = 2, \ldots, J \qquad \xi_1 = 0) \tag{1}$$

Starting from these coefficients we want to apply a transformation to obtain the coefficients that satisfy the sum to 0 constraints:

$$\xi_j = \mu + \alpha_j \qquad (\sum_j \alpha_j = 0) \tag{2}$$

Equalizing equation 1 and 2 when $j = 1$ we obtain:

$$\alpha_1 = -\mu$$

Equalizing equation 1 and 2 when $j > 1$ we obtain:

$$\alpha_j = \alpha_1 + \beta_j$$

Finally μ can be obtained equalizing $\sum_j \xi_j$ for the two constraints:

$$\begin{aligned} \sum_j \xi_j &= J\mu && \text{From equation 1} \\ \sum_j \xi_j &= \sum_j \beta_j && \text{From equation 2} \\ \mu &= \frac{\sum_j \beta_j}{J} \end{aligned}$$
